# Anti-Ma2 Paraneoplastic Encephalitis and Testicular Cancer: When the Hypothalamus Whispers—A Case Report and Systematic Review with Emphasis on Hypothalamic-Endocrine Dysfunction

**DOI:** 10.3390/medsci14020175

**Published:** 2026-03-31

**Authors:** Virginia Zamponi, Piero Paravani, Rossella Mazzilli, Flaminia Russo, Marina Paola Gardiman, Bruno Giometto, Raffaele Iorio, Alessandro Peri, Marco Zoccarato, Antongiulio Faggiano

**Affiliations:** 1 Endocrinology Unit, Department of Clinical and Molecular Medicine, Sapienza University of Rome, 00185 Rome, Italy; virginia.zamponi@uniroma1.it (V.Z.); piero.paravani@uniroma1.it (P.P.); rossella.mazzilli@uniroma1.it (R.M.); flaminia.russo@uniroma1.it (F.R.); 2 Surgical Pathology and Cytopathology Unit, Department of Medicine, University of Padua, 35122 Padua, Italy; marinapaola.gardiman@aopd.veneto.it; 3 Neurology Unit, Hospital of Trento, Azienda Provinciale per i Servizi Sanitari-APSS, 38122 Trento, Italy; bruno.giometto@apss.tn.it; 4 Department of Neuroscience, Università Cattolica del Sacro Cuore, 38122 Rome, Italy; raffaele.iorio@policlinicogemelli.it; 5 Pituitary Diseases and Sodium Alterations Unit, Endocrinology, Careggi University Hospital, University of Florence, 50121 Florence, Italy; alessandro.peri@unifi.it; 6 Neurology Unit, Ospedale Sant’Antonio, Azienda Ospedale Università di Padova, 35128 Padua, Italy; marcozoccarato@gmail.com

**Keywords:** paraneoplastic limbic encephalitis, anti-Ma2 antibodies, testicular germ cell tumor, hypothalamic dysfunction, central diabetes insipidus

## Abstract

**Background:** Paraneoplastic limbic encephalitis (PLE) with anti-Ma2 antibodies is a rare immune-mediated disorder associated with testicular cancer, particularly in young males. While neurological manifestations are well documented, hypothalamic–pituitary dysfunctions remain underreported. We present a case of anti-Ma2 PLE associated with testicular cancer together with a systematic review of PLE associated with testicular cancer, selectively restricted to anti-Ma2 positive cases and focusing on hypothalamic–endocrine involvement. **Case presentation:** We describe a 21-year-old male diagnosed with anti-Ma2 PLE and intratubular germ cell neoplasia of the right testis. He underwent orchifunicolectomy and immunosuppressive therapy with neurological improvement. Four years later, he developed new-onset temporal seizures, decreased libido, and a polyuria–polydipsia syndrome. Dynamic endocrine testing, including a water deprivation test and copeptin measurement, supported a diagnosis of partial central diabetes insipidus (CDI). **Methods:** A systematic literature review was performed in accordance with PRISMA guidelines. PubMed was searched using predefined keywords without time restriction. Studies reporting PLE associated with testicular tumors in humans with confirmed anti-Ma2 antibody positivity were included. **Results:** Eleven studies were included, reporting a total of 38 patients with anti-Ma2-associated PLE and testicular cancer. Hypothalamic or diencephalic involvement was described in 16 patients (42.0%), while endocrine manifestations were explicitly reported in four cases. Only two previous reports mentioned CDI, without detailed diagnostic evaluation. **Conclusions:** This study highlights the importance of recognizing hypothalamic-endocrine manifestations in PLE. In patients presenting with polydipsia and polyuria, CDI should be carefully differentiated from primary polydipsia using dynamic testing. Hypothalamic involvement may emerge years after tumor treatment, warranting long-term endocrine surveillance.

## 1. Introduction

Paraneoplastic neurological syndromes (PNSs) are a group of rare disorders that can affect both the central and the peripheral nervous system at various levels. They are related to effects not directly mediated by the tumor and its metastases, or by metabolic alterations, vascular, or infectious complications [1]. PNSs recognize an immune-mediated pathogenesis, and in many cases specific antibodies can be detected. They develop in approximately 1 out of 300 patients with cancer [2]. The most frequently encountered syndromes involve the central nervous system, including limbic encephalitis, subacute cerebellar degeneration, and paraneoplastic encephalomyelitis [2].

Paraneoplastic limbic encephalitis (PLE) is characterized by the subacute onset of short-term memory impairment neuropsychiatric symptoms, and temporal seizures [3], and may precede the diagnosis of the underlying malignancy by several months [4]. 

In some patients, the onset may be insidious, with depression or hallucinations that complicate the differential diagnosis with primary psychiatric disorders. Beyond classical limbic involvement, PLE, particularly in association with anti-Ma2 antibodies, may extend to diencephalic and hypothalamic structures, leading to disturbances of sleep–wake regulation, thermoregulation, appetite, and autonomic function.

The pathogenesis of PLE involves an immune response directed against onconeural antigens expressed both by tumor cells and by neurons within the central nervous system [1]. Small cell lung cancer (SCLC), testicular cancer, and breast cancer are the malignancies most frequently associated with PLE [5]. Notably, anti-Ma2-associated PLE shows a marked predilection for young males with testicular germ cell tumors and has been repeatedly associated with hypothalamic involvement, although endocrine consequences remain poorly characterized in the literature.

We present a case report and a systematic review of anti-Ma2-associated paraneoplastic limbic encephalitis in patients with testicular cancer, with particular attention to hypothalamic involvement and endocrine manifestations.

## 2. Material and Methods

A systematic review of the literature for the PLE associated with testicular cancer was performed according to the Preferred Reporting Items for Systematic Reviews and Meta-Analyses (PRISMA) guidelines. The systematic review was retrospectively registered on the Open Science Framework (OSF; registration ID: https://osf.io/nc2am).

### 2.1. Article Identification

The PubMed database was systematically searched from database inception to October 2025. The search strategy combined Medical Subject Headings (MeSH) and free-text terms related to paraneoplastic limbic encephalitis and testicular neoplasms. The exact PubMed search strategy was as follows:

(“paraneoplastic limbic encephalitis” OR “paraneoplastic encephalitis” OR “anti-Ma2 encephalitis”) AND (“testicular cancer” OR “testicular tumor” OR “testicular neoplasm” OR “germ cell tumor” OR “germ cell neoplasia”).

We included only English-language studies on humans, without time restriction, including randomized clinical trials, non-randomized trials, retrospective studies, and case reports. Review articles were also screened to identify additional relevant publications.

The inclusion criteria were: (1) articles reporting PLE associated with testicular cancer; (2) availability of histological confirmation of testicular neoplasia; (3) availability of neuronal antibody testing with confirmed anti-Ma2 antibody positivity; (4) studies on humans with full-text articles available. The exclusion criteria were: (1) non-English-language articles; (2) studies without histological confirmation; (3) studies without available full text; (4) cases with neuronal antibodies other than anti-Ma2 or with antibody status not specified.

Hypothalamic involvement was defined as the presence of clinical symptoms (e.g., hypersomnia, thermoregulation abnormalities, weight changes, dysautonomia) and/or neuroimaging findings affecting hypothalamic or diencephalic structures. Endocrine manifestations were defined as reported abnormalities of hypothalamic–pituitary axis function, including disorders of water balance (polyuria–polydipsia syndrome or diabetes insipidus), hypogonadism, thyroid dysfunction, adrenal insufficiency, or other hormonal disturbances described in the original reports. The definition of PLE was based on clinical criteria requiring at least two of the following three: (1) subacute onset (rapid progression over less than 3 months) of working memory deficits, seizures, or psychiatric symptoms; (2) bilateral brain abnormalities on T2-weighted fluid-attenuated inversion recovery (FLAIR) MRI restricted to the medial temporal lobes; and (3) at least one of the following: CSF pleocytosis or EEG showing epileptic or slow-wave activity involving the temporal lobes [6].

### 2.2. Article Selection

Three authors independently screened articles by title and abstract (Virginia Zamponi, Rossella Mazzilli, Piero Paravani). Disagreements between reviewers were resolved through discussion between the two reviewers. If consensus was not reached, a third senior author (A.F.) independently reviewed the study and made the final decision. Eligible studies were subsequently assessed through full-text review. Data extraction was independently performed by three authors (Virginia Zamponi, Piero Paravani, Marco Zoccarato).

A standardized data extraction form was used to collect the following information: first author, year of publication, study design, number of reported patients, clinical presentation, tumor laterality, histological findings, antibody profile, neuroimaging features, therapeutic strategies, neurological outcomes, and evidence of hypothalamic–pituitary dysfunction. When available, information regarding antibody detection methods (e.g., Western blot, immunoblot, immunohistochemistry) and sample source (serum and/or CSF) was also extracted. Antibody titers were not reported in the majority of the included studies and were therefore not included in the analysis.

The initial search identified 194 records. After removal of duplicates and records excluded before screening, 143 records were screened by title and abstract. After screening titles and abstracts, 51 articles were selected for full-text evaluation. Following full-text review, 18 articles were considered potentially eligible. Of these, six articles were excluded because patients did not show anti-Ma2 antibody positivity or antibody status was not clearly specified.

**Figure 1:**
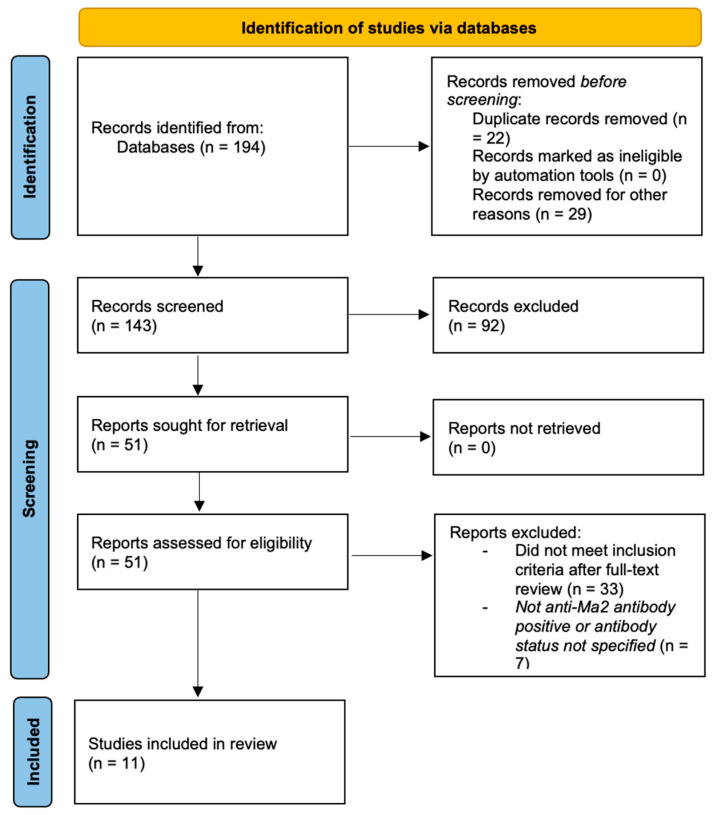
Flow diagram of the study selection process for the systematic review.

A total of 11 articles met all inclusion criteria and were included in the final analysis. These studies collectively reported 38 patients with anti-Ma2-associated PLE and testicular cancer. The study selection process is illustrated in a PRISMA 2020 flow diagram [7] (Figure 1).

## 3. Case Report

**Figure 2:**
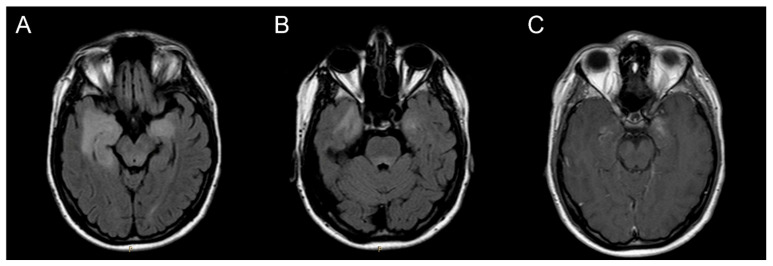
FLAIR bilateral mesial temporal hyperintensity involving the mesial temporal lobes (**A**) and the periventricular and dorsal area of the pons (**B**). Gadolinium-enhanced MRI sequence showing bilateral enhancement (**C**).

In November 2015, a 21-year-old man with no significant past medical history presented with acute onset of confusion, irritability and anterograde amnesia. The patient also had pain in the right groin. A brain MRI showed bilateral inflammation and swelling in the mesial temporal lobes and periventricular and dorsal areas of the mesencephalon and pons (Figure 2).

**Figure 3:**
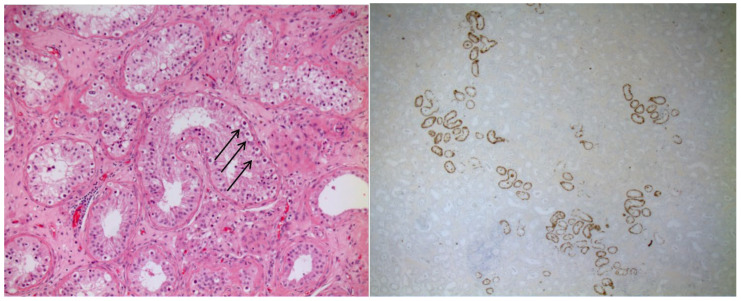
LEFT (H&E). Seminiferous tubules showing germ cell neoplasia in situ with atypical gonocytes (arrows) characterized by clear cytoplasm and large nuclei. RIGHT. PLAP immunohistochemistry shows germ cell neoplasia in situ.

Analysis of the cerebrospinal fluid (CSF) showed a normal cell count, a mildly elevated protein level and the presence of intrathecal oligoclonal bands. An extensive search for infectious causes of encephalitis was negative. However, screening for onconeural antibodies by commercial immunofluorescence and immunoblotting revealed anti-Ma2 antibody positivity. Immunofluorescence was performed using serum (1:10) and cerebrospinal fluid (CSF; undiluted, 1:1), whereas immunoblotting was performed using serum (1:100) and CSF (1:4). Screening for neuronal surface antibodies was negative. A whole-body 18FDG PET/CT showed some reactive lymph nodes in the abdomen, and a testicular ultrasound showed a small 7 mm hypoechoic area in the upper pole of the right testis. The patient was diagnosed with PLE and treated firstly with intravenous methylprednisone (1 g for 5 days), and then with oral prednisone (50 mg/day). Intravenous immunoglobulin (IVIG) was planned with monthly administration of 2 g/kg over four days. Given the persistence of cognitive features, the patient underwent surgical orchifunicolectomy in May 2016. Pathological examination of the testis revealed intratubular germ cells neoplasia (IGCN) (Figure 3).

The patient continued on oral prednisone and monthly cycles of IVIG for a further three months, resulting in improvement in the cognitive and behavioral changes. The recovery was successful and allowed the patient to pursue high-level studies. In the years following surgery, the patient reported good health, with the exception of a mild memory deficit, and no significant morbid events or trauma.

**Figure 4:**
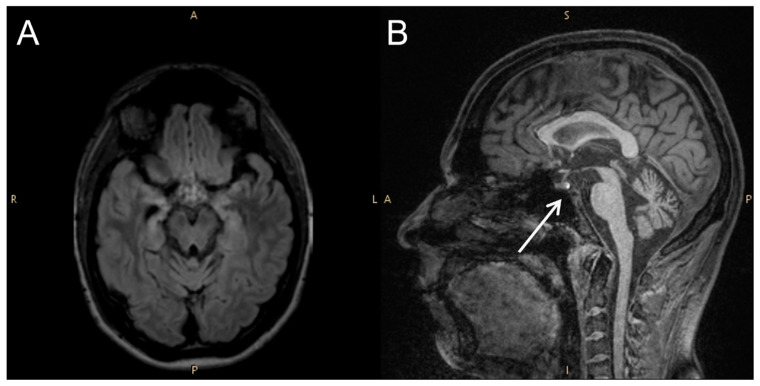
Follow-up brain MRI. Axial FLAIR image (**A**) showing bilateral atrophy of the hippocampus and amygdala. Sagittal T1 image (**B**) showing the normal hyperintense appearance of the neurohypophysis (arrow).

Four years after orchiectomy, the patient began to experience temporal epileptic seizures, characterized by a gasp, speech arrest and subsequent retrograde amnesia lasting several hours; these episodes occurred more frequently during the waking phase. The seizures were only partially responsive to antiepileptic drugs, including valproic acid and lamotrigine. This drug-refractory epilepsy persisted with the same characteristics over a 4-year follow-up period. In addition, the patient reported polyuria, polydipsia (drinking 6 litres of water per day), and decreased sexual desire with erectile dysfunction. Brain MRI showed atrophic involvement of mesial temporal structures and normal findings in the brainstem and hypothalamic region. Notably, the usual hyperintensity in T1 sequences of neurohypophysis was maintained (Figure 4).

Cerebrospinal fluid examination was normal, except for the persistence of anti-Ma2 both in the CSF and in the serum. During the endocrinological outpatient evaluation, the patient underwent a comprehensive assessment. Clinical examination revealed a body mass index (BMI) of 30.3 kg/m^2^, blood pressure of 140/80 mmHg, and no evidence of oedema. Scrotal ultrasound of the left testicle demonstrated normal testicular volume without detectable lesions. Seminal analysis indicated that all parameters exceeded the 5th percentile according to the WHO 2021 guidelines [8]. Hormonal profiling showed values within the normal range (Table 1). Water homeostasis parameters revealed a serum sodium concentration of 136 mmol/L, plasma osmolality of 277 mOsmol/kg H_2_O, and urinary osmolality of 140 mOsmol/kg. Additional laboratory analyses showed serum calcium concentrations of 2.32 mmol/L and potassium of 4.2 mmol/L. Serum copeptin concentration was 1.9 pmol/L, which excluded a diagnosis of nephrogenic diabetes insipidus.

**Figure 5:**
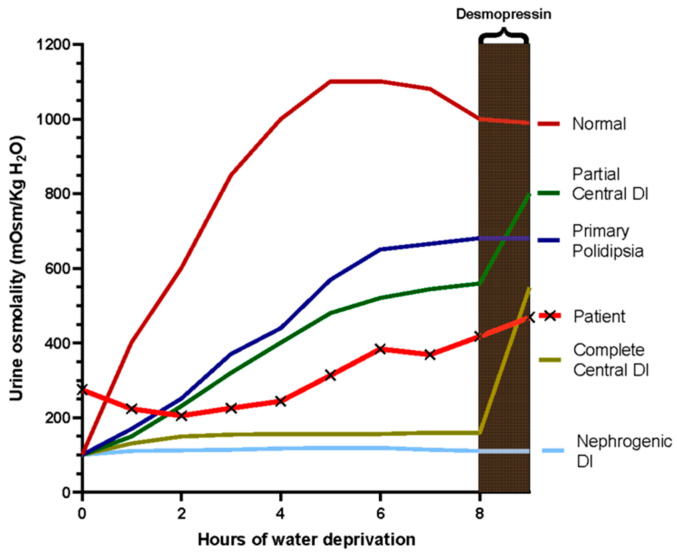
Nomogram of the trend of urinary osmolarity during the thirst test in the reported case as compared to normal and different types of pathological pattern.

For the suspicion of central diabetes insipidus (CDI) probably related to late-onset hypothalamic involvement due to paraneoplastic encephalitis, the patient underwent a water deprivation test (WDT) (Table 2). The WDT was performed under medical supervision with periodic monitoring of body weight, blood pressure, heart rate, urine volume, urine osmolality, plasma sodium concentration, and plasma osmolality. Measurements were obtained at regular intervals during the dehydration phase. The test was discontinued after approximately eight hours of dehydration due to stabilization of urinary osmolality and progressive body weight reduction. Subsequently, desmopressin (4 µg intramuscularly) was administered and urine osmolality was reassessed to evaluate the renal response. In our case, after eight hours of water deprivation, the urine osmolality reached 418 mOsm/kg (Figure 5 and Table 2). For differential diagnosis between primary polydipsia and partial CDI, desmopressin was administered and caused an increase in urine osmolality concentration >9%, but less than 50% (from 418 to 469, 12%), indicating a state of partial CDI (Figure 5).

## 4. Review

### 4.1. Patients and Clinical Symptoms

In the cohort of 38 patients with PLE associated with testicular cancer and anti-Ma2 antibodies, the most frequently reported clinical manifestations were those attributable to limbic system involvement. Specifically, 33 patients (86.8%) presented with symptoms such as anterograde amnesia, memory disturbances, confusion, disorders of consciousness, and disorientation. These features are characteristic of limbic dysfunction and were frequently accompanied by seizures (reported in 10 cases, 26,3%), further supporting the limbic localization of the encephalitic process. Psychiatric disturbances were also frequent, including anxiety (n = 3), irritability (n = 2), depression (n = 2), panic attacks (n = 1), mutism (n = 1), obsessive–compulsive symptoms (n = 1), and non-specific psychiatric disturbances (n = 1).

Extralimbic involvement is frequently reported. In particular clinical signs of brainstem dysfunction were described in 16 patients (42.1%), with manifestations such as dysarthria, diplopia and/or extrinsic oculomotor dysfunction, gait and balance deterioration and/or ataxia suggesting involvement of cerebellar and pontine structures. Neuroimaging findings often corroborated these clinical signs, indicating a broader encephalitic distribution.

Symptoms referable to diencephalic or hypothalamic involvement were reported in 16 patients (42,1%). These included weight gain, hypersomnia or sleepiness, hypokinesia, lethargy, narcolepsy/cataplexy, dysautonomia, thermoregulatory abnormalities, and headache. These manifestations reflect disruption of homeostatic, circadian, and neuroendocrine circuits regulated by the hypothalamus and its connections.

Suspected endocrinological abnormalities, potentially suggestive of hypothalamic–pituitary axis involvement, were described in 4 patients, sometimes with multiple concurrent alterations. The described findings included reduced libido, hypogonadism, hypothyroidism, and polyuria–polydipsia syndromes. Given the heterogeneous and often incomplete diagnostic workup, these alterations could not be consistently attributed to a specific endocrine etiology. Notably, two patients were reported to have CDI, although diagnostic details were not uniformly available across reports.

The neurological manifestations in the reviewed cohort are summarized in Table 3.

### 4.2. Presenting Symptoms and Timing

In most analysed cases (31 out of 38), neurological symptoms represented the initial clinical manifestation, preceding the diagnosis of the underlying testicular neoplasm. In this group, the median interval between the onset of neurological disturbances and cancer diagnosis was six months, with a minimum latency of two months and a maximum of 36 months. This pattern highlights the importance of recognizing early neurological signs as potential paraneoplastic indicators, particularly in young male patients.

Conversely, in the remaining eight patients, PLE developed after the diagnosis of testicular cancer had already been established. In these cases, the latency between tumor diagnosis and the emergence onset of neurological symptoms was more variable, with a median duration of 12 months. The shortest interval was one month, while the longest reported latency—168 months (14 years) suggests the possibility of delayed autoimmune activation or tumor recurrence with new paraneoplastic expression.

These findings underscore the variability in PLE onset relative to the neoplastic process and emphasize the importance of long-term neurological monitoring in patients with a history of testicular germ cell tumors.

### 4.3. Neuroimaging

Neuroimaging data were available for 27 of the 38 patients included in this review. Among these, 21 patients showed radiological abnormalities on brain MRI, while imaging was reported as normal in the remaining five. In a subset of patients with abnormal MRI findings, however, the precise topographical distribution of the lesions was not specified, limiting detailed anatomical interpretation.

Overall, limbic system involvement was the most frequently reported radiological pattern, observed in 14 patients, confirming the selective vulnerability of these structures in PLE. Typical findings included temporal lobe signal abnormalities on T2/FLAIR sequences, variably involving the hippocampus, amygdala, or adjacent mesial temporal structures, with either unilateral or bilateral distribution.

Diencephalic and hypothalamic involvement was identified in six patients, occasionally extending to adjacent basal ganglia structures. Reported abnormalities included lesions affecting the hypothalamus, thalamus (including the pulvinar), pallidum, and mammillary bodies, reflecting the anatomical continuity of limbic–diencephalic networks.

Brainstem involvement was described in five patients, with lesions variably affecting the periaqueductal gray matter, pons, or midbrain. In a smaller subset, cerebellar involvement was documented (two patients), most commonly in the form of cerebellar atrophy.

These radiological patterns reinforce the concept of preferential autoimmune targeting of limbic and diencephalic structures in PLE, while also highlighting the potential for multifocal central nervous system involvement.

The neuroimaging findings in the reviewed cases are summarized in Table 4.

### 4.4. Testicular Imaging and Tumor Laterality

Among the 38 patients reviewed, testicular ultrasound findings were available for 12 individuals. A solid intratesticular lesion was identified in three cases, while testicular microcalcifications were reported in six patients. In two additional cases, microcalcifications were associated with a concomitant solid intratesticular mass. In one patient, ultrasound examination revealed isolated hydrocele. Regarding laterality, 9 abnormalities were in the right testis, while three were bilateral; no cases of isolated left-sided involvement were reported.

These data suggest a predominance of right-sided testicular involvement, although bilateral abnormalities were also observed. This highlights the importance of comprehensive bilateral scrotal imaging in patients with suspected PLE, particularly in the context of supportive neurological and serological findings. Notably, Mathew et al. reported three cases in which uni- or bilateral testicular microcalcifications (at the first evaluation or at the follow up) were the only ultrasonographic finding, and yet a prophylactic orchiectomy revealed IGCN on histological examination [9]. This underscores the diagnostic value of considering surgical exploration in selected cases, even in the absence of clearly defined testicular masses (see below). Testicular ultrasound results are presented in Table 5.

### 4.5. Histology

Among the 17 patients for whom histological data were available, the testicular cancer histotype was reported with sufficient detail to allow classification. The neoplastic entity most frequently associated with PLE was IGCN, identified in eight patients. This lesion is widely recognized as a precursor to invasive germ cell tumors and underscores the importance of early detection and timely intervention.

Mixed germ cell tumors were reported in two patients. These included combinations of different germ cell components, reflecting the pluripotent nature of germ cell malignancies and their potential association with paraneoplastic neurological syndromes.

In six cases, histopathological examination confirmed the presence of a germ cell tumor but did not provide further histological subclassification. These cases were therefore classified as germ cell tumors not otherwise specified (NOS).

Notably, pure teratoma was exceptionally rare in this context; only one case, reported by Hoffmann et al., described PLE associated with an isolated testicular teratoma.

Histological findings of the testicular tumors are summarized in Table 6.

### 4.6. Therapy and Outcomes

Therapeutic data were available for all 38 patients included in this review. Management strategies addressed both the underlying testicular neoplasm and the associated PLE, often through multimodal approaches.

Orchiectomy represented the cornerstone of oncological management and was performed in 34 of the 38 patients, confirming its central role in the treatment of testicular cancer-associated PLE. In several cases, orchiectomy was undertaken despite the absence of a clearly defined testicular mass, particularly in the presence of microcalcifications or subclinical ultrasound abnormalities, supporting the concept of early removal of the presumed antigenic source. In particular, Mathew et al. reported six cases of anti-Ma2 encephalitis in which unilateral or bilateral orchiectomy was made on the basis of life threatening symptoms, young age, absence of other tumors and presence of new testicular enlargement or risk factors for germ-cell tumors, mainly cryptorchidism or ultrasound evidence of testicular microcalcifications [9]. Histological examination revealed IGCN in all the cases [9].

In a subset of patients undergoing orchiectomy, systemic chemotherapy and/or radiotherapy were administered according to tumor characteristics and disease stage, in line with standard management of testicular germ cell tumors. These oncological treatments were frequently combined with neurological supportive care.

With regard to the management of PLE, immunomodulatory therapies—including high-dose corticosteroids, plasmapheresis, and intravenous immunoglobulin (IVIG)—were employed in selected patients, particularly in those with severe, rapidly progressive, or disabling neurological symptoms. Importantly, immune-directed treatments were used in addition to tumor-directed therapy, rather than as standalone interventions, reflecting a combined oncological–immunological therapeutic strategy. In isolated refractory cases, second-line immunotherapies, such as rituximab, were administered [6]

Symptomatic treatments were commonly required. Antiepileptic drugs were widely used to control seizures, while other agents, including antidepressants and central nervous system stimulants such as dexamphetamine, were prescribed in selected cases to manage neuropsychiatric symptoms and disorders of vigilance.

Neurological outcome data were available for 22 patients. Among these, nine patients experienced neurological improvement or partial remission following treatment. Eight patients remained neurologically stable without substantial recovery, while two patients showed progressive neurological deterioration despite therapy.

Fatal outcomes occurred in four patients during follow-up.

Overall, these findings highlight the heterogeneity of neurological outcomes in anti-Ma2-associated PLE and underscore the potential impact of combined tumor-directed and immunomodulatory treatment strategies on disease course.

## 5. Discussion

This case, together with the present systematic review, illustrates several distinctive features of anti-Ma2 PLE-associated with testicular germ cell neoplasia. As in most published reports, the patient initially presented with acute limbic dysfunction—confusion, irritability, and short-term memory impairment—which preceded the diagnosis of the underlying neoplasm [4,10,11,12]. This pattern is well recognized in anti-Ma2 PLE, particularly in young males, and highlights the importance of early neurological recognition in cases of unexplained subacute amnesia or behavioral change.

Neuroimaging findings in our patient, characterized prominently by bilateral mesial temporal abnormalities are consistent with previously described radiological patterns involving the limbic system [9,12,13,14,15]. Atrophy and mesial temporal sclerosis developing gradually after the acute phase, as observed in our case, along with the development of drug-resistant epilepsy, can be related to chronic immune-mediated neuronal injury [10,11]. These findings underscore the potential for long-term structural sequelae and the need for extended neurological follow-up.

A central contribution of this case concerns hypothalamic–pituitary involvement. Although clinical manifestations suggestive of hypothalamic dysfunction, such as hypersomnia, temperature dysregulation, weight gain, were documented in a substantial proportion of published cases [11,12,16], explicit endocrine abnormalities were reported far less frequently.

Only a few reports described suspected endocrine-related abnormalities such as hyponatremia, libido changes, hypogonadism, hypocortisolism, or polyuria–polydipsia syndromes [10,15,16]. Only two cases briefly mentioned CDI, without providing sufficient diagnostic detail.

Our case is the first to provide a comprehensive and dynamic endocrine evaluation for suspected late-onset CDI in the context of anti-Ma2 PLE. Four years after the acute episode, the patient developed persistent polyuria–polydipsia and sexual dysfunction—features compatible with hypothalamic involvement. The combination of water deprivation testing, desmopressin response, and basal copeptin measurement supported a diagnosis of partial CDI.

The differential diagnosis of polyuria–polydipsia syndrome primarily includes CDI (complete or partial), nephrogenic diabetes insipidus, and primary polydipsia. In the present case, nephrogenic diabetes insipidus was considered unlikely because basal copeptin levels were low (1.9 pmol/L), whereas patients with nephrogenic diabetes insipidus typically exhibit elevated copeptin concentrations due to preserved vasopressin secretion in the presence of renal resistance. The main alternative diagnosis was therefore primary polydipsia. During the water deprivation test, urinary osmolality progressively increased but remained below the values expected in normal subjects (>600–800 mOsm/kg), suggesting impaired urinary concentrating ability. Following desmopressin administration, urinary osmolality increased from 418 to 469 mOsm/kg (+12%), a response compatible with partial CDI rather than primary polydipsia, in which little or no additional increase is typically observed after exogenous vasopressin analogues. It should be acknowledged that the distinction between partial CDI and primary polydipsia may remain challenging in some cases, particularly when stimulated copeptin testing is not performed. Nevertheless, in the present case the combination of low basal copeptin levels, incomplete urinary concentration during dehydration, and a modest but measurable response to desmopressin supports the diagnosis of partial CDI. In addition, the known predilection of anti-Ma2-associated encephalitis for diencephalic and hypothalamic structures provides a plausible pathophysiological substrate for impaired vasopressin regulation.

Delayed hypothalamic or diencephalic manifestations, emerging months or years after tumor treatment, have been described in scattered reports [11,12,14] and suggest that autoimmune activity may persist or re-emerge long after tumor treatment. Our case expands this concept by showing that such delayed manifestations may also include subtle yet clinically significant disruptions of water homeostasis.

Taken together, the available evidence suggests that hypothalamic–endocrine involvement in anti-Ma2 PLE may be more frequent than currently appreciated, although it remains insufficiently characterized in the literature. This peculiar pattern of involvement, together with the frequent occurrence in young male patients and the strong association with testicular germ cell tumors, clearly differentiates this subgroup from other forms of paraneoplastic limbic encephalitis associated with neuronal antibodies such as anti-Hu, anti-CV2, anti-AMPAR, and anti-GABABR, which are most commonly linked to small cell lung cancer [21].

PLE is likely underrecognized. Given the frequency of hypothalamic symptoms across published cases and the rarity of structured endocrine assessment, clinicians should maintain a high index of suspicion for CDI and pituitary dysfunction in patients presenting with polyuria, polydipsia, fatigue, or sexual symptoms—even years after apparent neurological stabilization. Systematic endocrine surveillance may therefore aid in early detection and improve long-term outcomes.

## 6. Conclusions

This case report and systematic review highlight that hypothalamic involvement in anti-Ma2 PLE is more frequent than previously recognized, yet endocrine consequences remain underreported. Our case represents the first documented instance of delayed-onset partial CDI supported by dynamic endocrine testing. Clinicians should be aware that hypothalamic–pituitary dysfunction may emerge years after tumor treatment, and systematic endocrine surveillance, particularly in patients with polyuria, polydipsia, or sexual dysfunction, should be considered to improve long-term outcomes.

## Figures and Tables

**Table 1:** Values of Hormonal Assays for Laboratory Analysis.

Serum Parameter (Unit)	Value	Normal Range
Testosterone (nmol/L)	12.84	9.7–38.1
FSH (UI/L)	4.57	0.95–11.95
LH (UI/L	3.43	0.57–12.1
PRL (mIU/L)	459.6	145–790
SHBG (nmol/L)	18.30	13.5–71.4
TSH (mIU/L)	1.63	0.35–4.0
FT4 (pmol/L)	15.48	9.0–19.0
ACTH (pmol/L)	4.93	1.04–11.3
Cortisol (nmol/L)	308.8	133–538

**Table 2:** Water deprivation test.

Time (h)	BP (mmHg)	HR (bpm)	Weight (Kg)	Urine (cc)	Natremia (mmol/L) v: 136–145	pOsm (mOsm/L) v: 275–295	uOsm (mOsm/L) v: 400–1100
7:30	140/80	70	101.5	0	135	267	275
8:30	130/80	70	101	500	134	262	224
9:30	130/80	68	101	400	135	263	205
10:30	130/80	68	100.7	350	135	264	225
11:30	120/80	68	100.4	400	137	264	244
12:30	110/70	70	100	200	NA	262	314
13:30	110/70	68	99.7	120	137	263	384
14:30	110/70	68	99.6	100	138	264	369
15:30	120/75	68	99.5	NA	137	263	418
16:30	100/75	68	99.2	60	137	265	469

h = Hours; BP = Blood Pressure; HR = Heart Rate; IM = intramuscular; NA = not available.

**Table 3:** Neurological manifestations according to the anatomical region involved [9,10,11,12,13,14,15,16,17,18,19,20].

Study	Study Type	Patients (n)	Limbic Symptoms (n)	Hypothalamic-Diencephalic Symptoms (n)	Brainstem Symptoms (n)
Chen, C., 2021 [13]	Case report	1	1	0	0
Voltz, R., 1999 [11]	Case series	10	8	5	4
Landolfi, J.C., 2003 [12]	Case report	1	1	1	0
Kimura, M., 2008 [10]	Case report	1	1	1	0
Pfister, Ch., 2003 [17]	Case report	1	1	0	0
Mathew, R.M., 2007 [9]	Case series	6	6	3	4
Suwijn, S.R., 2016 [16]	Case report	1	0	1	0
Matsumoto, L., 2007 [14]	Case report	1	1	0	1
Lei, Y., 2022 [15]	Case report	1	1	1	1
Hoffmann, L.A., 2008 [19]	Case series	4	3	1	1
Dalmau, J., 2004 [20]	Case series	11	9	3	5

**Table 4:** Reports the radiological findings, focusing on involvement of limbic, hypothalamic–diencephalic, brainstem, and cerebellar regions [9,10,11,12,13,14,15,16,17,18,19,20].

Study	Study Type	Patients (n)	MRI Available (n)	MRI Normal (n)	Limbic Symptoms (n)	Hypothalamic-Diencephalic Symptoms (n)	Brainstem Symptoms (n)	Cerebellum (n)
Chen, C., 2021 [13]	Case report	1	1	0	1	0	0	0
Voltz, R., 1999 [11]	Case series	10	10	3	1	NR	NR	NR
Landolfi, J.C., 2003 [12]	Case report	1	1	0	1	0	0	0
Kimura, M., 2008 [10]	Case report	1	1	0	1	1	0	0
Pfister, Ch., 2003 [17]	Case report	1	1	0	1	0	0	0
Mathew, R.M., 2007 [9]	Case series	6	5	1	3	3	2	1
Suwijn, S.R., 2016 [16]	Case report	1	1	0	1	1	0	0
Matsumoto, L., 2007 [14]	Case report	1	1	0	1	1	1	0
Lei, Y., 2022 [15]	Case report	1	1	0	1	1	1	0
Hoffmann, L.A., 2008 [19]	Case series	4	4	1	3	0	0	0
Dalmau, J., 2004 [20]	Case series	11	0	NR	NR	NR	NR	NR

Abbreviations: MRI = Magnetic Resonance Imaging; NR = Not Reported.

**Table 5:** Detection methods of anti-Ma2 antibodies, testicular ultrasound findings, and tumor laterality reported in case reports and case series of patients with anti-Ma2 PLE [9,10,11,12,13,14,15,16,17,18,19,20]. Minor ultrasound abnormalities include findings such as microlithiasis, microcalcifications, hydrocele, or subtle parenchymal changes without a clear focal mass.

Study	Study Type	Patients (n)	Technique of Detection of MA2 abs	US Available	Suspicious Lesion	Minor US Abnormalities	Right	Left	Bilateral
Chen, C., 2021 [13]	Case report	1	NR	1	1	0	1	0	0
Voltz, R., 1999 [11]	Case series	10	WB, IHC	0	NR	NR	NR	NR	NR
Landolfi, J.C., 2003 [12]	Case report	1	WB, IHC	1	0	1	1	0	0
Kimura, M., 2008 [10]	Case report	1	NR	1	1	0	1	0	0
Pfister, Ch., 2003 [17]	Case report	1	IHC	0	NR	NR	NR	NR	NR
Mathew, R.M., 2007 [9]	Case series	6	IB	6	1	6	4	0	2
Suwijn, S.R., 2016 [16]	Case report	1	NR	1	0	1	1	0	0
Matsumoto, L., 2007 [14]	Case report	1	NR	1	0	1	0	0	1
Lei, Y., 2022 [15]	Case report	1	NR	1	1	0	1	0	0
Hoffmann, L.A., 2008 [19]	Case series	4	WB, ICH	0	NR	NR	NR	NR	NR
Dalmau, J., 2004 [20]	Case series	11	WB, IHC	0	NR	NR	NR	NR	NR

Abbreviations: NR = not reported; US = ultrasound; WB = Western blot; IB = immunoblot; IHC = immunohistochemistry.

**Table 6:** Histological findings of testicular tumors reported in case reports and series of patients with anti-Ma2 PLE [9,10,11,12,13,14,15,16,17,18,19,20].

Study	Study Type	Patients (n)	Histology Available (n)	Germ Cell	Teratoma	Mixed	IGCN
Chen, C., 2021 [13]	Case report	1	1	1	0	0	0
Voltz, R.,1999 [11]	Case series	10	0	NR	NR	NR	NR
Landolfi, J.C., 2003 [12]	Case report	1	1	0	0	0	1
Kimura, M., 2008 [10]	Case report	1	1	0	0	1	0
Pfister, Ch., 2003 [17]	Case report	1	1	1	0	0	0
Mathew, R.M., 2007 [9]	Case series	6	6	0	0	0	6
Suwijn, S.R., 2016 [16]	Case report	1	1	1	0	0	0
Matsumoto, L., 2007 [14]	Case report	1	1	0	0	0	1
Lei, Y., 2022 [15]	Case report	1	1	0	0	1	0
Hoffmann, L.A., 2008 [19]	Case series	4	4	3	1	0	0
Dalmau, J., 2004 [20]	Case series	11	0	NR	NR	NR	NR

Abbreviations: NR = not reported; IGCN = Intratubular Germ Cell Neoplasia.

## Data Availability

The original contributions presented in this study are included in the article. Further inquiries can be directed to the corresponding author.
